# Neuro-ophthalmic complications of modern anti-cancer drugs

**DOI:** 10.1007/s00417-023-06350-4

**Published:** 2024-02-12

**Authors:** Joshua A. Oskam, Helen V. Danesh-Meyer

**Affiliations:** 1 School of Optometry and Vision Science, Faculty of Medical and Health Sciences, University of Auckland, Private Bag 92019, Auckland, 1142 New Zealand; 2 Department of Ophthalmology, Greenlane Clinical Centre, Auckland, Auckland, New Zealand; 3 Department of Ophthalmology, Faculty of Medical and Health Sciences, University of Auckland, Auckland, New Zealand

**Keywords:** Neuro-ophthalmology, Targeted cancer therapy, Complications

## Abstract

**Purpose:**

Targeted cancer therapies have been responsible for a dramatic shift in treatment strategies for cancer, and the number of drugs, classes, and indications are continually growing. Neuro-ophthalmic complications of these medications are an uncommon but important subset of adverse events which profoundly impact vision. This review aims to collate studies and reports of known neuro-ophthalmic complications of targeted therapies and describe their management.

**Methods:**

The anti-cancer drugs included in the review were any drugs targeting specific molecules involved in the cancer disease process. PubMed, EMBASE, and Web of Science were searched using the generic names of each drug and keywords of neuro-ophthalmic conditions. The prescribing information published by the US Food and Drug Administration (FDA) for each drug was also reviewed.

**Results:**

Several classes of targeted anti-cancer drugs were found to cause neuro-ophthalmic adverse effects. Immune checkpoint inhibitors are responsible for a raft of immune-related adverse events such as optic neuritis, ischemic optic neuropathy, PRES, and myasthenia gravis. Therapies with anti-VEGF activity can provoke posterior reversible leukoencephalopathy, which commonly presents with visual loss and can be fatal if not treated promptly. Inhibitors of BCR-ABL1, VEGF, ALK, and proteasomes have all been linked to optic nerve disorders which can have debilitating consequences for vision.

**Conclusion:**

The neuro-ophthalmic complications of modern anti-cancer drugs can limit or necessitate the withdrawal of these life-prolonging medications. Ophthalmologists should be alert for neuro-ophthalmic complications in these medications to facilitate prompt diagnosis and treatment and reduce the risk of severe and permanent consequences.


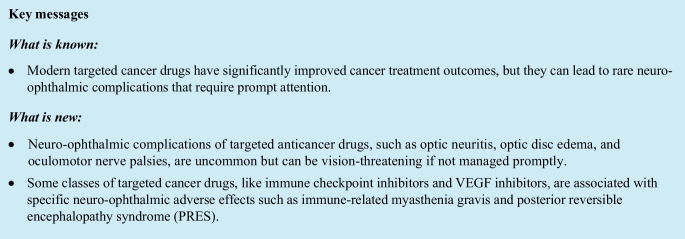


## Introduction

Modern, targeted cancer drugs have revolutionized the treatment of cancer over the past two decades and contributed to the continued progress in improving cancer survival rates. The field is rapidly expanding with increasing numbers of drugs developed with broadening indications. Despite their importance in treatment strategies for cancer, these drugs are not without complications. Several classes are known to cause a range of ocular adverse effects—most of which can be managed with specific treatments without ceasing the cancer therapy. Specifically, neuro-ophthalmic adverse effects are uncommon but are vision-threatening if not detected and treated promptly and may herald serious involvement of other organ systems. This review seeks to amalgamate and discuss neuro-ophthalmic complications of modern anti-cancer drugs and their management.

## Method

The anti-cancer drugs included in the review of publications were any drugs targeting specific molecules involved in the cancer disease process, namely tyrosine kinase inhibitors, immune checkpoint inhibitors, proteasome inhibitors, antibody–drug conjugates, and selective inhibitors of nuclear export. PubMed, EMBASE, and Web of Science were searched using the generic names of each drug and the following keywords: neuro-ophthalmic, optic neuropathy, optic neuritis, optic disc edema, hypophysitis, diplopia, ptosis, oculomotor nerve palsy, trochlear nerve palsy, abducens nerve palsy, myasthenia gravis, posterior reversible encephalopathy syndrome, and giant cell arteritis. Hormonal anti-cancer drugs were excluded, and results were restricted to the English language. For adverse effects involving other organ systems (e.g., myasthenia gravis) only articles where there was explicit ocular involvement were included. The prescribing information published by the US Food and Drug Administration (FDA) for each drug was also reviewed for any information regarding neuro-ophthalmic adverse effects. A summary of the drugs and their neuro-ophthalmic complications is included in Table 1.

**Table 1:** Summary of targeted anti-cancer drugs and neuro-ophthalmic complications

Class	Medication	Common FDA-approved indications	Neuro-ophthalmic complications
BCR-ABL1 inhibitors	Imatinib	CML, ALL, GIST	Optic neuritis, optic neuropathy, optic disc edema, myasthenia gravis
	Dasatinib	CML, ALL	Optic neuritis, optic neuropathy, optic disc edema
VEGF inhibitors	Bevacizumab	Colorectal cancer, NSCLC, glioblastoma, cervical cancer, RCC	Optic neuritis, optic neuropathy, PRES, 6th nerve palsy, 3rd nerve palsy
	Apatinib	Adenoid cystic carcinoma	PRES
ALK inhibitors	Crizotinib	NSCLC	Visual disturbances, optic neuropathy
	Ceritinib	NSCLC	Visual disturbances
	Lorlatinib	NSCLC	Visual disturbances
	Entrectinib	NSCLC	Visual disturbances
	Alectinib	NSCLC	Visual disturbances
Proteasome inhibitors	Bortezomib	Multiple myeloma, mantle cell lymphoma	Optic neuropathy, 3rd nerve palsy, PRES
	Carfilzomib	Multiple myeloma	PRES
BRAF inhibitors	Vemurafenib	Melanoma	PRES, myasthenia gravis
	Dabrafenib	Melanoma, NSCLC	PRES, myasthenia gravis
HER2 inhibitors	Trastuzumab	Breast cancer, gastric cancer	PRES
Multikinase inhibitors	Sunitinib	GIST, RCC, pancreatic neuroendocrine tumor	Optic neuritis, optic neuropathy, optic disc edema, PRES, EOM disorders
	Pazopanib	RCC, soft tissue sarcoma	Optic neuritis, optic neuropathy, optic disc edema, PRES, EOM disorders
	Sorafenib	HCC, RCC, thyroid cancer	PRES, EOM disorders
	Regorafenib	Colorectal cancer, GIST	PRES
	Lenvatinib	Thyroid cancer, RCC, HCC, endometrial carcinoma	PRES
Immune checkpoint inhibitors			
- CTLA-4 inhibitors	Ipilimumab	Melanoma	Optic neuritis, MG, GCA, PRES, INO, 6th nerve palsy, 3rd nerve palsy, opsoclonus-myoclonus-ataxia syndrome
	Tremelimumab	HCC	
- Anti-PD-1 inhibitors	Nivolumab	Melanoma, NSCLC, SCLC, RCC, cHL, HNSCC, urothelial carcinoma, colorectal cancer, HCC	
	Pembrolizumab	Melanoma, NSCLC, SCLC, HNSCC, cHL, urothelial carcinoma, gastric cancer, cervical cancer, RCC	
	Cemiplimab	NSCLC, cutaneous SCC, NSCLCC	
- Anti-PD-L1 inhibitors	Atezolizumab	Melanoma, NSCLC, urothelial carcinoma, HCC	
	Durvalumab	NSCLC, urothelial carcinoma	
	Avelumab	Merkel cell carcinoma	

*ALL* acute lymphocytic leukemia, *cHL* classical Hodgkin’s lymphoma, *CML* chronic myeloid leukemia, *EOM* extraocular muscle, *GCA* giant cell arteritis, *GIST* gastrointestinal stromal tumour, *HCC* hepatocellular carcinoma, *HNSCC* head and neck squamous cell carcinoma, *INO* internuclear ophthalmoplegia, *MG* myasthenia gravis, *NSCLC* non-small cell lung cancer, *PRES* posterior reversible encephalopathy syndrome, *RCC* renal cell carcinoma, *SCLC* small cell lung cancer

## Results

### Breakpoint cluster region-Abelson 1 (BCR-ABL1) inhibitors

BCR-ABL1 is a fusion protein and product of the Philadelphia (Ph) chromosome, a translocation between chromosomes 9 and 22. It activates a tyrosine kinase that leads to proliferation and malignant transformation of hematopoietic cells in chronic myeloid leukemia (CML) and is also present in many cases of acute lymphoblastic leukemia (ALL) [1].

Imatinib, a BCR-ABL1 inhibitor, was approved by the FDA in 2001 and was the first small-molecule tyrosine kinase inhibitor (TKI) to be used in cancer, paving the way for future targeted therapies. While most commonly directed at Ph + CML and ALL) [2], it also demonstrates activity against c-kit receptor and platelet-derived growth factor (PDGF) receptor mutations, to which end, it is also utilized against gastrointestinal stromal tumors (GIST) [3].

The FDA prescribing information for imatinib states that optic neuritis is a rare complication (0.01%–0.1%) [2]. Despite its 20-year history, only four case reports exist in the literature of optic neuritis, and five cases of optic disc edema [4–11]. The time of onset from treatment initiation was extremely variable in these cases—from 1 month [5] to 9 years [9]—with a similarly variable severity of presentation. Treatment cessation alone was sufficient to treat cases of optic disc edema, while optic neuritis responded to conventional treatment with corticosteroids, including one patient who made a full recovery from counting fingers vision in both eyes [5]. Rarely, more aggressive treatment was required. A case report described a 35-year-old man who developed combined optic neuritis and transverse myelitis, with bilateral vision loss and retro-orbital pain, as well as weakness, sensory loss, and urinary retention [6]. There was no response to systemic steroids initially, so the patient underwent seven treatments of plasma exchange and ceased imatinib, with a gradual full recovery of vision and strength. Another patient presented with unilateral optic neuritis and MRI lesions typical for demyelination but retained normal vision [6]. Treatment was not stopped, but after further lesions appeared on follow-up MRI the patient commenced disease-modifying therapy.

Dasatinib is a second-generation TKI approved for the treatment of Ph + CML and ALL [12]. Similarly, cases have been reported with optic disc edema, toxic optic neuropathy and optic neuritis [7, 13, 14]. A patient taking dasatinib developed optic neuropathy with bilateral superior arcuate scotomas after 2.5 months of therapy [13]. Dasatinib was withdrawn and the patient treated with high dose oral prednisone. There was only modest improvement in the visual fields at 6 months after treatment. In a case of optic neuritis which progressed to near blindness in one eye, dasatinib was not stopped until a year after onset when symptoms appeared in the contralateral eye. Visual recovery was achieved in the better eye with corticosteroids; however, symptoms recurred after switching to imatinib [7]. Bosutinib, another BCR-ABL inhibitor, was then trialed without incident. A multi-center retrospective review of 109 cases of CML treated with dasatinib found one case complicated by papilledema, although no further detail is available [14].

PDGF receptor inhibition has been proposed as a mechanism by which BCR-ABL1 inhibitors induce optic nerve edema and optic neuritis. The drug class is particularly associated with fluid retention, including periorbital edema which occurs in 24.2%–80% of patients taking imatinib [4, 15]. Inhibition of PDGF receptors decreases interstitial pressure and increases transcapillary fluid transport—conceivably, the mechanism through which edema occurs, and this may extend to the optic nerve tissue [16]. Furthermore, PDGF (together with insulin) prevents apoptosis of retinal ganglion cells through the PI 3-kinase/Akt pathway [17]. Biswas observed that imatinib disrupts PDGF receptor signaling leading to apoptotic cell death in rat retinal ganglion cells [17]—therefore, optic disc edema and optic neuritis potentially result from either increased interstitial fluid flow, apoptosis of retinal ganglion cells, or a combination of both.

In a solitary case report imatinib appeared to cause or “unmask” myasthenia gravis in a patient treated with imatinib for GIST [18]. Symptoms included ptosis with onset a few days after initiating treatment and recurrence on re-challenge 2 days later. Anti-MuSK antibodies were positive with a high titer. The patient’s condition deteriorated dramatically requiring intensive care support, pyridostigmine, plasma exchange, intravenous immunoglobulin (IVIG), and steroid therapy—eventually making a full recovery. The authors note that imatinib inhibits immunosuppressive regulatory T cells, an effect also seen in myasthenia gravis related to thymoma [19].

### Vascular endothelial growth factor (VEGF) inhibitors

VEGF is a cytokine secreted by tumor cells promoting endothelial cell proliferation and angiogenesis at the tumor site [20]. Anti-VEGF therapies target this angiogenesis but commonly encounter resistance to treatment, indicating there are more complex and overlapping pathways involved [20].

VEGF inhibitors are associated with posterior reversible encephalopathy syndrome (PRES) – a condition characterized by headaches, vision loss (usually homonymous hemianopia), decreased mental state and seizures, with characteristic MRI findings of vasogenic edema in the occipital and parietal areas of the brain bilaterally [21]. PRES is also linked to solid organ transplantation, pre-eclampsia, autoimmune disorders, and the use of traditional chemotherapy drugs and immunosuppressants [21]. Patients are usually but not necessarily hypertensive at diagnosis—hypertension being an established complication of VEGF inhibitors, likely via reduced production of nitric oxide and other mechanisms [22]. A review of published cases of PRES secondary to anti-VEGF agents found that it occurred more commonly in female patients, and that proteinuria—another adverse effect of anti-VEGF therapy—was present whenever tested for [23]. The exact pathophysiology behind PRES is still debated, the most prevalent theories arguing that hypertension causes hyperperfusion and endothelial damage, or that vasoconstriction leads to hypoperfusion and brain ischemia [24]. Over-secretion of anti-diuretic hormone secondary to anti-VEGF therapy has also been raised as a potential mechanism [25]. Treatment is generally supportive and requires prompt removal of the offending anti-cancer treatment, management of hypertension, and treatment and prevention of seizures [21]. Visual symptoms occur in 17.6% to 40% of patients with PRES as a consequence of anti-VEGF therapy, and visual decline can be precipitous [23, 26]. It is therefore important that ophthalmologists consider the diagnosis in patients on VEGF inhibitors with visual impairment and a normal ocular examination.

Bevacizumab is a VEGF inhibitor indicated for the treatment of colorectal cancer, non-small cell lung cancer, glioblastoma, cervical cancer, and renal cell carcinoma [27]. In a single-center, retrospective review of incidences of PRES in patients with cancer, 6 of the 31 identified patients had received bevacizumab and 2 had been treated with both bevacizumab and sunitinib, a multikinase inhibitor with anti-VEGF activity [28]. This represented 0.1% of all patients who had received systemic bevacizumab treatment for cancer over the same period. Eight of the 31 patients with PRES (of any cause) experienced visual disturbance. 84% recovered completely in a median of 7.5 days after treatment cessation and supportive measures where indicated. The remaining patients had progression of cancer as a possible explanation for persistent reduced mental state or died of unrelated causes.

Nine case reports of PRES with bevacizumab describe visual symptoms at presentation, including cortical blindness, visual impairment, and diplopia [29–38]. Eight made a full recovery with drug discontinuation and supportive measures alone; however, one patient died [32]. PRES has also been reported occasionally in more recently developed anti-VEGF agents. A case of PRES occurred in a patient treated with apatinib—a selective VEGFR-2 inhibitor—who presented with blurred vision and diplopia [39]. There was a complete recovery after the drug was held, and it was safely restarted at a reduced dose.

Several cases of optic neuropathy have been identified in patients with glioblastoma treated with bevacizumab. A retrospective review found 6 such patients out of 503 total (1.2%) developed severe optic neuropathy progressing to complete vision loss in one or both eyes [40]. Examination findings of the optic discs were not reported; however, enhancement of the affected optic nerves was sometimes seen on MRI T1 or FSE T2. All had also received fractionated radiation therapy and temozolomide. Bevacizumab has not been associated with optic neuropathy when treating other malignancies, and the authors suggest a possible priming effect from radiation therapy. One case of unilateral retrobulbar optic neuritis has been reported in a patient treated with bevacizumab and docetaxel for breast cancer, leading to total blindness in the affected eye [35]. Two further cases of optic neuritis are mentioned as adverse reactions in clinical trials [41, 42].

One report each of abducens nerve [43] and pupil-involving oculomotor nerve [44] palsies have been tied to the use of systemic bevacizumab. Both cases were transient: the patient with abducens nerve palsy fully recovered 3 months after bevacizumab was discontinued, while the patient with oculomotor nerve palsy was additionally treated with methylprednisolone and achieved resolution in 2 weeks. Investigations did not reveal another cause and the pathophysiology remains unclear.

### Anaplastic lymphoma kinase (ALK) inhibitors

A small portion of non-small-cell lung cancers (NSCLC) involve activating rearrangements of the ALK gene, the most common of which is the EML4-ALK fusion gene (2 to 7% of all NSCLC), the target of ALK inhibitors [45].

Crizotinib is an ALK and ROS1 inhibitor approved for NSCLC and anaplastic large cell lymphoma [46]. Visual disturbances and photopsias are its most common adverse effect, occurring in 60–64% of patients [47, 48]. These have been described as lights trailing objects, more noticeable with changes in ambient lighting from dark to light and generally mild and unobtrusive [45]. ALK is expressed in the nerve fiber layer of the retina, but its precise function remains unknown. Crizotinib was found to reduce b-wave amplitude on electroretinogram in rats; however, this was not replicated in rats treated with lorlatinib, another ALK inhibitor, indicating some other mechanism may be at play [49].

The FDA prescribing information for crizotinib states that 0.2% of 1719 patients across all clinic trials suffered Grade 4 visual field defects and cited optic atrophy and optic nerve disorders as potential causes [46]. One case report of optic neuropathy has been described [50]. A 69-year-old patient on crizotinib therapy for NSCLC with brain metastases developed transient visual shadows followed by blindness in one eye and superior hemifield loss in the contralateral eye after 3 months of treatment. An MRI of the brain revealed enhancing optic nerves bilaterally which were not present on previous imaging. Crizotinib was withheld but there was no amelioration of symptoms. It was restarted 3 months later with renewed progression of visual field deficit in the seeing eye. While the authors consider crizotinib to be the most likely cause, the mechnanism is unestablished. Prescribing information for more recently approved ALK-inhibitors—ceritinib, lorlatinib, entrectinib, and alectinib—report vision disorders in 4.6% to 21% of patients, including blurred vision, photopsia, and diplopia [51–54].

### Proteasome inhibitors

The ubiquitin–proteasome pathway is responsible for the degradation and turnover of intracellular proteins and is key to many cell processes including the cell cycle and apoptosis [55]. Proteasome inhibitors were developed with the intention of targeting cachexia in cancer patients by slowing protein degradation; however, they were found to also induce apoptosis of cancer cells in mouse models [55].

Bortezomib was the first proteasome inhibitor to be developed and is indicated for the treatment of multiple myeloma and mantle cell lymphoma [56]. A case series described two patients with optic atrophy deemed likely secondary to bortezomib [57]. Both cases presented with gradual deterioration in vision over months with visual field defects, optic disc pallor and thinning; one also exhibited mild peripheral neuropathy. Discontinuation of bortezomib only provided a modest improvement in vision. Another report instead linked bortezomib to the onset of a unilateral oculomotor nerve palsy during the first cycle of therapy in a 54-year-old woman with multiple myeloma [58]. Symptoms improved with treatment delay and dexamethasone but deteriorated again after re-challenge with bortezomib. Treatment was discontinued and dexamethasone re-trialed but without further improvement. These appear to be the only reported cases to date and are without a clear mechanism; however, peripheral neuropathy is a common adverse effect of bortezomib, usually manifesting as distal neuropathic pain [59]. The process by which this occurs is also undetermined but is likely to include proinflammatory cytokines and epigenetic changes elicited by bortezomib [59].

PRES secondary to bortezomib and carfilzomib, a second-generation proteasome inhibitor, has been reported rarely [60–64]. One patient developed ocular apraxia and simultagnosia without field loss, in addition to reduced vision [63]. All cases resolved promptly with supportive measures and drug discontinuation. The link between proteasome inhibitors and PRES is unknown, although inhibition of the proteasome reduces activation of transcription factor nuclear factor-kappa B and therefore several growth factors, including VEGF—which could feasibly be the responsible mechanism by which PRES is caused, as described in the section “VEGF inhibitors” [61].

### BRAF inhibitors

The B-Raf protein, encoded by the BRAF gene, plays an important role in regulating cell proliferation via the Ras/Raf/MEK/ERK/MAP pathway [65]. Oncogenic BRAF mutations are present in around 66% of melanomas and to a lesser extent in some other cancers [65]. BRAF inhibitors have been occasionally linked to PRES—by increasing levels of cytokines, including tumor necrosis factor-α and interferon-γ, that increase vascular permeability and potentially lead to cerebral edema [66].

Vemurafenib was the first BRAF inhibitor to be approved and is indicated in melanomas with BRAF V600E mutations [67]. One case of presumed PRES with blurred vision has been reported in a patient taking vemurafenib, although the diagnosis could not be confirmed as MRI was not performed [68]. Symptoms resolved over a week with antihypertensives and cessation of vemurafenib, though they later recurred when vemurafenib was restarted. Dabrafenib, another BRAF inhibitor, resulted in a case of PRES with Bálint’s syndrome—a triad of optic ataxia, oculomotor apraxia, and simultanagnosia [69]. The patient was not hypertensive, but MRI was suggestive of immune-mediated meningitis in addition to PRES, so high-dose corticosteroids were started and tapered off. Symptoms slowly improved and did not recur when the patient was switched to vemurafenib.

Both vemurafenib and dabrafenib have been implicated in rare cases of myasthenia gravis, more traditionally associated with immune checkpoint inhibitors. A patient with metastatic melanoma developed myasthenia gravis with ptosis after 2 weeks of dabrafenib and trametinib, an MEK inhibitor. Treatment was paused and symptoms were managed with pyridostigmine, but recurred when the drugs were restarted [70]. A similar presentation occurred in a patient treated with combination vemurafenib and cobimetinib, another MEK inhibitor [18]. Zaloum et al. point to evidence that inhibition of the BRAF and MEK enzymes possibly unbalance immune signaling and cytokine expression, which may be the underlying mechanism in these cases [70, 71].

### Human epidermal growth factor 2 (HER2) inhibitors

Approximately 20–30% of breast cancers exhibit an overexpression of HER2 receptor tyrosine kinase, which is associated with a more aggressive clinical course and poorer survival [72]. Trastuzumab was developed in the 1990s to target HER2-positive breast cancers and had a dramatic impact on cancer response rates and survival. Use was later extended to HER2-positive metastatic gastric cancer.

One case of PRES secondary to trastuzumab has been reported in a 54-year-old woman treated for gastric cancer, in combination with cisplatin and capecitabine. Symptoms included vision loss occurring after the fourth cycle of treatment, which resolved rapidly after antihypertensives and treatment discontinuation. Trastuzumab is known to downregulate VEGF [73] and can cause hypertension as an adverse effect [74]: the underlying mechanism may therefore be similar to that proposed for PRES caused by anti-VEGF agents.

### Multikinase inhibitors

Multikinase inhibitors target a broad range of tyrosine kinases important in cancer therapy and have a correspondingly wide range of adverse effects. The neuro-ophthalmic complications reported in association with multikinase inhibitors include optic neuritis/neuropathy (possibly via PDGF inhibition), and PRES (likely via VEGF inhibition). A database review of spontaneous adverse event reports for selected oral anti-VEGF drugs (all multikinase inhibitors) yielded an incidence of 19 cases of optic disc edema or ischemic optic neuropathy out of 691 total ocular adverse events for patients taking sunitinib, 3 out of 278 ocular adverse events for those taking pazopanib and none for those taking sorafenib [75]. Small numbers of extraocular muscle disorders including ptosis were reported for all three drugs, but without further detail and no other cases were found in the literature. Beyond this study, a case of optic neuritis secondary to sunitinib was reported which resolved with corticosteroids and drug discontinuation [76], while another patient with optic disc edema, blurred vision and diplopia due to sunitinib achieved a full recovery with treatment cessation alone [77]. A patient taking cabozantinib developed bilateral optic disc edema with decreased vision, which only partially improved after steroid therapy and the drug was ceased [78].

Cases of PRES with visual symptoms have been reported in patients treated with sunitinib [79–86] pazopanib [87–90], sorafenib, [91, 92] regorafenib [93], and lenvatinib [94]. All drugs demonstrate activity against VEGF receptors, among several other tyrosine kinases. Bilateral reduced visual acuity was the most common complaint, and in some cases as severe as total loss of light perception. Also reported were visual field defects, hemifield visual neglect, amaurosis fugax, visual hallucinations, and optic ataxia. With drug cessation and supportive treatment all cases saw complete resolution within hours to weeks.

### Immune checkpoint inhibitors

Immune checkpoints are molecules expressed on a broad array of immune cells and are key to regulating immune responses. Their upregulation in tumor cells leads to evasion of anti-tumor T-cells and encourages tumor immune escape [95]. Immune checkpoint inhibitors (ICIs) are a rapidly expanding class of monoclonal antibodies utilized against an increasing range of cancers. Current drugs in clinical use inhibit the immune checkpoint molecules of T cells—specifically anti-cytotoxic T lymphocyte antigen 4 (CTLA-4), programmed cell death 1 (PD-1) or its ligand (PD-L1) [96]. The over-stimulation of the immune system in this way can bring with it various unwanted “immune-related adverse events”—behaving similarly to autoimmune diseases—which impact multiple organ systems and at times prove fatal [97]. However, the occurrence of immune-related adverse events may also be correlated with greater cancer response [98, 99].

Ocular toxicity secondary to ICIs is uncommon, having been estimated to occur in 2.8—3.6% of patients, with the most frequent reactions being dry eye and uveitis [100, 101]. Neuro-ophthalmic complications occur with a 1-year incidence of 1.3–1.4%, and patients with a history of a neuro-ophthalmic diagnosis or uveitis have an increased chance of recurrence while taking immune checkpoint inhibitors [102]. The relatively low proportion of ophthalmic side effects compared to other organ systems may owe itself to the anatomical and physiological immune privilege of the eye [103].

Ipilimumab, a CTLA-4 inhibitor, was the first ICI to be approved by the FDA in 2011, for use in melanoma [104]. It is responsible for the highest rate of immune-related adverse events among individual ICI drugs, likely due to the more proximal location where CTLA-4 functions in the pathway of immune system activation, compared to PD-1/PD-L1 [105]. Tremelimumab is another anti-CTLA-4 antibody, currently under investigation for several cancers [106]. Anti-PD-1 inhibitors with reported neuro-ophthalmic complications include pembrolizumab, nivolumab, and cemiplimab, while the anti-PD-L1 inhibitors are atezolizumab, avelumab, and durvalumab. Anti-CTLA-4 and anti-PD-1 combination therapy provokes more immune-related adverse events than anti-PD-1 monotherapy [107–109]. Systemic corticosteroids are the mainstay of treatment for immune-related adverse effects. Dosage and duration of treatment are extremely variable between cases—reflecting a lack of treatment guidelines—however, they are generally tailored to the severity and natural history of the adverse effect [110]. Milder cases can at times permit ICIs to be continued or held and restarted with judicious use of corticosteroids, although this may dampen tumor response.

Optic nerve disorders, including optic disc edema, toxic optic neuropathy, and optic neuritis, comprise a small number of reported immune-related adverse effects of ICIs [111]. Symptoms usually consist of blurred vision and visual field defects: as a distinction from classical optic neuritis, pain is an uncommon symptom. A small subset of cases of hypophysitis also present with visual involvement [112, 113]. Treatment involves IV and/or oral high-dose corticosteroids in almost all patients; while plasma exchange, IVIG and other immunosuppressants/steroid-sparing agents are occasionally required. Visual outcome is generally favorable, and Sun et al. suggest that the decision to stop, withhold or continue the immune checkpoint inhibitor be based on severity of visual impairment and response to treatment [111].

Myasthenia gravis and myasthenic syndromes are one of the more common immune-related adverse events that occur with ICI treatment, with an incidence of around 0.1–0.47% of total immune-related adverse events [114–117]. Unusually, myasthenia also has a higher reported incidence in anti-PD-1/PD-L1 ICIs compared to anti-CTLA-4 (ROR 3.9) [100]. Median time to onset of symptoms is approximately 1 month, and ptosis and diplopia are common presenting symptoms [100, 103–105]. ICI-related myasthenia exhibits some important differences compared to the idiopathic type: it is generally more severe, with a quicker deterioration and higher rates of bulbar symptoms and myasthenic crisis [102, 103]. Acetylcholine receptor antibodies are more often at lower levels and are undetectable in half of patients, making accurate diagnosis challenging [103, 106–108]. It also frequently co-presents as an overlap syndrome with myositis and/or myocarditis, which bring with them a higher mortality rate and should prompt cautious screening in patients with symptoms of myasthenia [109–111]. A systematic review of cases with this triad syndrome found that 60% of patients died in hospital from the acute presentation and complications [111]. Most cases are treated with steroid therapy, and many receive anticholinesterase drugs: intravenous immunoglobulin and plasma exchange may improve outcomes when used as first-line therapy in addition to steroids [117].

Sporadic case reports have been published of giant cell arteritis (GCA) secondary to ICIs with ocular symptoms, in patients between 62 and 88 years old [118–122]. The time between treatment initiation and onset of symptoms ranged from a week after the first dose to 1 year. Ocular symptoms were typical for giant cell arteritis and included amaurosis fugax, transient diplopia, blurry vision, and unilateral total visual loss. Treatment with high-dose corticosteroids saw improvement or resolution in all cases but one who suffered a central retinal artery occlusion and remained at no light perception in the affected eye [122]. No cases specifically describe ischemic optic neuropathy. Cadena et al. identified that GCA is a likely a result of age-related changes in blood vessels as well as the immune system, including the CTLA-4 and PD-1 immune checkpoint pathways [123]. Blocking and releasing these checkpoints in a susceptible, older population may provoke GCA, a theory supported by the role of abatacept, a novel treatment for GCA that contains CTLA-4 and inhibits CD28-mediated T cell co-stimulation [124].

Six cases of PRES secondary to ICI use have been reported [125–130]. All responded well to standard supportive treatment, although one patient was left with persistent visual hallucinations [127]. Sabile et al. reported on a patient who developed PRES after switching from combination ipilimumab and nivolumab to encorafenib (a BRAF inhibitor) and binimetinib (a MEK inhibitor), which raised the difficulty of determining the precipitating agent [128]. The authors argue that ICI therapy is the most likely cause of PRES in this instance, or the consecutive use of an ICI then BRAF/MEK inhibitors. There are no reported cases of PRES with encorafenib, although a few cases have occurred secondary to other BRAF inhibitors, as described above. The mechanism by which ICIs may cause PRES has not been determined; however, about half of patients with PRES have a history of autoimmune disease [131]. Stimulation of the immune system by ICIs, accompanied by increased inflammatory cytokines may lead to endothelial activation and damage with subsequent edema [125, 131].

Other, rare neuro-ophthalmic complications include internuclear ophthalmoplegia [132, 133] and oculomotor and abducens nerve palsies [133–136]. A case of Tolosa-Hunt syndrome secondary to ipilimumab improved symptomatically with corticosteroids and local radiation but little change in vision was achieved [137]. A case of opsoclonus-myoclonus-ataxia syndrome (although without myoclonus) saw resolution with corticosteroids, IVIG, clonazepam and sodium valproate [137].

## Discussion

The advent and rapid expansion of targeted cancer therapies has brought about improved cancer survival for a vast array of malignancies, but not without the potential for complications. Neuro-ophthalmic complications are rare but when they occur risk significant and permanent visual consequences. At times, ocular signs and symptoms signal potentially fatal conditions such as myasthenia gravis. Some complications exhibit a strong medication class effect, such as immune-related adverse events and ICIs, while others, such as PRES and optic neuritis, are linked to multiple classes. It is important to note, however, that in many instances such complications are only documented in case reports, so conclusions cannot necessarily be drawn on their association with the treatment drug, especially in conditions also known to be paraneoplastic disorders. While measures such as corticosteroids can treat many of these complications, drug discontinuation is usually required and this has important implications for an individual’s cancer treatment and prognosis, requiring multi-disciplinary discussion. It is of key importance that ocular symptoms are promptly referred and investigated, and that ophthalmologists are aware of the possibility of neuro-ophthalmic complications in patients with a history of treatment with targeted anti-cancer drugs. Furthermore, certain patients may be at higher risk of developing neuro-ophthalmic complications, such as those with a history of neuro-ophthalmic or autoimmune conditions taking ICIs, and this should be taken into account by oncologists when discussing treatment selection.
